# 2,2′-Biimidazolium hexa­aqua­manganese(II) bis­(sulfate)

**DOI:** 10.1107/S1600536808020291

**Published:** 2008-07-09

**Authors:** Mukhtar A. Kurawa, Christopher J. Adams, A. Guy Orpen

**Affiliations:** a School of Chemistry, University of Bristol, Bristol BS8 1TS, England

## Abstract

The title compound, (C_6_H_8_N_4_)[Mn(H_2_O)_6_](SO_4_)_2_, was obtained by cocrystallization of 2,2′-biimidazolium sulfate and bis­(tetra­butyl­ammonium) tetra­chlorido­manganate(II). The asymmetric unit contains one isolated (SO_4_)^2−^ anion, one half of an octa­hedral [Mn(H_2_O)_6_]^2+^ dication and one half of a 2,2′-biimidazolium dication, each of which lies on an inversion centre. Mol­ecules are connected by a three-dimensional N—H⋯O and O—H⋯O hydrogen-bond network.

## Related literature

For the syntheses, structural studies and thermal behaviour of related compounds, see: Rekik *et al.* (2006 ▶, 2007 ▶).
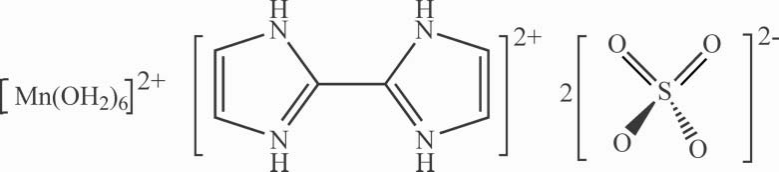


## Experimental

### 

#### Crystal data

(C_6_H_8_N_4_)[Mn(H_2_O)_6_](SO_4_)_2_
*M* *_r_* = 491.34
Monoclinic, 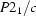

*a* = 6.0625 (7) Å
*b* = 11.606 (2) Å
*c* = 12.218 (2) Å
β = 91.65 (1)°
*V* = 859.3 (2) Å^3^
*Z* = 2
Mo *K*α radiation
μ = 1.09 mm^−1^
*T* = 100 (2) K
0.4 × 0.3 × 0.2 mm

#### Data collection

Bruker SMART APEX CCD area-detector diffractometer
Absorption correction: multi-scan (*SADABS*; Sheldrick,1996 ▶) *T*_min_ = 0.686, *T*_max_ = 0.800
9389 measured reflections
1954 independent reflections
1897 reflections with *I* > 2σ(*I*)
*R*_int_ = 0.018

#### Refinement

*R*[*F* ^2^ > 2σ(*F* ^2^)] = 0.023
*wR*(*F* ^2^) = 0.066
*S* = 1.06
1954 reflections
142 parameters
6 restraints
H atoms treated by a mixture of independent and constrained refinement
Δρ_max_ = 0.28 e Å^−3^
Δρ_min_ = −0.65 e Å^−3^

### 

Data collection: *SMART* (Bruker, 2007 ▶); cell refinement: *SAINT* (Bruker, 2007 ▶); data reduction: *SAINT*; program(s) used to solve structure: *SHELXS97* (Sheldrick, 2008 ▶); program(s) used to refine structure: *SHELXL97* (Sheldrick, 2008 ▶); molecular graphics: *SHELXTL* (Sheldrick, 2008 ▶); software used to prepare material for publication: *SHELXTL*.

## Supplementary Material

Crystal structure: contains datablocks I, global. DOI: 10.1107/S1600536808020291/hy2142sup1.cif

Structure factors: contains datablocks I. DOI: 10.1107/S1600536808020291/hy2142Isup2.hkl

Additional supplementary materials:  crystallographic information; 3D view; checkCIF report

## Figures and Tables

**Table 1:** Selected bond lengths (Å)

Mn1—O6	2.1335 (10)
Mn1—O7	2.1856 (10)
Mn1—O5	2.2218 (10)

Symmetry code: (i) 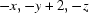.

**Table 2:** Hydrogen-bond geometry (Å, °)

*D*—H⋯*A*	*D*—H	H⋯*A*	*D*⋯*A*	*D*—H⋯*A*
N1—H1*A*⋯O3^ii^	0.88	1.93	2.7699 (15)	159
N1—H1*A*⋯O2^ii^	0.88	2.45	3.0994 (15)	131
N2—H2*A*⋯O3^iii^	0.88	1.92	2.7562 (16)	159
O5—H5*A*⋯O2	0.843 (14)	1.917 (15)	2.7600 (15)	176.8 (18)
O5—H5*B*⋯O3^iv^	0.813 (14)	2.088 (15)	2.8638 (15)	159.7 (17)
O6—H6*A*⋯O4^v^	0.839 (14)	1.908 (15)	2.7402 (15)	171.2 (18)
O6—H6*B*⋯O1^vi^	0.846 (14)	1.847 (15)	2.6904 (14)	174.2 (18)
O7—H7*A*⋯O4^vii^	0.851 (14)	1.888 (15)	2.7298 (15)	169.5 (18)
O7—H7*B*⋯O2^v^	0.846 (14)	1.892 (14)	2.7266 (14)	169.0 (17)

Symmetry codes: (ii) 
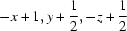
; (iii) 
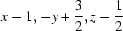
; (iv) 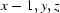; (v) 
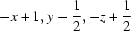
; (vi) 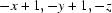; (vii) 
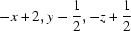
.

**Fig. 1.**
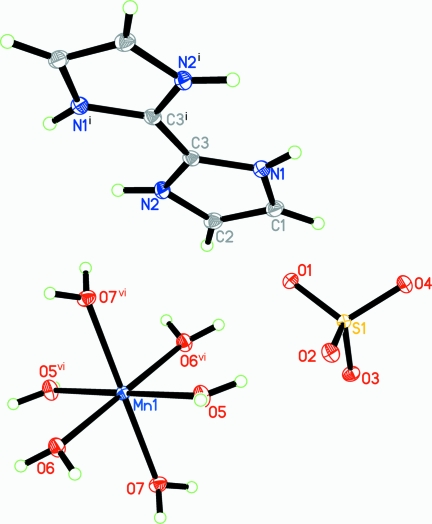
The molecular structure of the title compound. Displacement ellipsoids are drawn at the 50% probability level. [Symmetry codes: (i) -x, 2-y, -z; (vi) 1-x, 1-y, -z.]

**Fig. 2.**
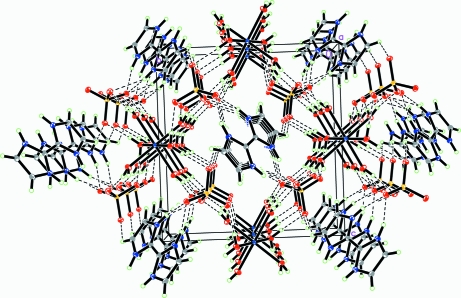
Packing diagram for the title compound viewed along the a-axis.

## supplementary crystallographic information

### Comment

The syntheses, structural studies and thermal behaviour of similar complexes with piperazinium, (C_4_H_12_N_2_)^2+^, and 1,4-diaza-bicyclo[2.2.2]octandiium, (C_6_H_14_N_2_)^2+^, cations have been reported (Rekik *et al.*, 2006, 2007).

In the crystal structure of the title compound (Fig. 1; Table 1), the (C_6_H_8_N_4_)^2+^, [Mn(H_2_O)_6_]^2+^ and (SO_4_)^2-^ ions are connected by N—H···O and O—H···O hydrogen bonds (Table 2), with the 2,2'-biimidazolium dications in the supramolecular cavities formed by the metal–sulfate framework (Fig. 2). The corresponding structures of some first row transition metal M^II^ sulfates (M = Mn, Ni, Fe and Cu) templated with piperazinium display similar three-dimensional hydrogen-bonded networks (Rekik, Naili, Bataille *et al.*, 2006). In particular, the structures of the (C_4_H_12_N_2_)^2+^[M(H_2_O)_6_]^2+^(SO_4_)_2_^2-^ (M = Mn or Ni) compounds contain channels (running parallel to the *c*-axis in those cases), which are defined by a square arrangement of [M(H_2_O)_6_]^2+^ cations and which contain the organic dications, mirroring the channels seen in the title compound (Fig. 2).

### Experimental

The title compound was obtained unintentionally as the product of an attempted synthesis of a hydrogen-bonded salt of the tetrachloromanganate(II) anion and the biimidazolium cation, using slow evaporation of a water–acetonitrile solution (1:1 v/v) of equimolar amounts of bis(tetrabutylammonium) tetrachloromanganate(II) and 2,2'-biimidazolium sulfate at room temperature.

### Refinement

H atoms bonded to O atoms were located in a difference map and refined with distance restraints of O—H = 0.84 (2) Å and with *U*_iso_(H) = 1.2*U*_eq_(O). Other H atoms were positioned geometrically and refined using a riding model, with C—H = 0.95 and N—H = 0.88 Å, and with *U*_iso_(H) = 1.2*U*_eq_(C, N).

### Crystal data

**Table:**

(C_6_H_8_N_4_)[Mn(H_2_O)_6_](SO_4_)_2_	*F*_000_ = 506
*M**_r_* = 491.34	*D*_x_ = 1.899 Mg m^−^^3^
Monoclinic, *P*2_1_/*c*	Mo *K*α radiation λ = 0.71073 Å
Hall symbol: -P 2ybc	Cell parameters from 7442 reflections
*a* = 6.0625 (7) Å	θ = 2.4–27.5º
*b* = 11.606 (2) Å	µ = 1.09 mm^−^^1^
*c* = 12.218 (2) Å	*T* = 100 (2) K
β = 91.65 (1)º	Block, colourless
*V* = 859.3 (2) Å^3^	0.4 × 0.3 × 0.2 mm
*Z* = 2	

### Data collection

**Table:**

Bruker SMART APEX CCD area-detector diffractometer	1954 independent reflections
Radiation source: fine-focus sealed tube	1897 reflections with *I* > 2σ(*I*)
Monochromator: graphite	*R*_int_ = 0.018
*T* = 100(2) K	θ_max_ = 27.5º
φ and ω scans	θ_min_ = 2.4º
Absorption correction: multi-scan(SADABS; Sheldrick,1996)	*h* = −7→7
*T*_min_ = 0.686, *T*_max_ = 0.800	*k* = −15→15
9389 measured reflections	*l* = −15→15

### Refinement

**Table:**

Refinement on *F*^2^	Secondary atom site location: difference Fourier map
Least-squares matrix: full	Hydrogen site location: inferred from neighbouring sites
*R*[*F*^2^ > 2σ(*F*^2^)] = 0.023	H atoms treated by a mixture of independent and constrained refinement
*wR*(*F*^2^) = 0.066	*w* = 1/[σ^2^(*F*_o_^2^) + (0.042*P*)^2^ + 0.4497*P*] where *P* = (*F*_o_^2^ + 2*F*_c_^2^)/3
*S* = 1.06	(Δ/σ)_max_ < 0.001
1954 reflections	Δρ_max_ = 0.29 e Å^−^^3^
142 parameters	Δρ_min_ = −0.65 e Å^−^^3^
6 restraints	Extinction correction: none
Primary atom site location: structure-invariant direct methods	

### Fractional atomic coordinates and isotropic or equivalent isotropic displacement parameters (Å^2^)

**Table:**

	*x*	*y*	*z*	*U*_iso_*/*U*_eq_	
Mn1	0.5000	0.5000	0.0000	0.00892 (9)	
S1	0.87954 (5)	0.76694 (3)	0.24181 (2)	0.00829 (10)	
N1	0.20621 (19)	1.00486 (9)	0.11407 (9)	0.0107 (2)	
H1A	0.1629	1.0584	0.1597	0.013*	
N2	0.21706 (17)	0.89187 (9)	−0.02676 (9)	0.0105 (2)	
H2A	0.1825	0.8585	−0.0896	0.013*	
C1	0.3926 (2)	0.93818 (11)	0.12742 (11)	0.0125 (2)	
H1B	0.4969	0.9413	0.1869	0.015*	
C2	0.3988 (2)	0.86702 (11)	0.03924 (11)	0.0124 (2)	
H2B	0.5082	0.8106	0.0257	0.015*	
C3	0.1021 (2)	0.97533 (11)	0.02099 (10)	0.0098 (2)	
O1	0.87996 (15)	0.78300 (8)	0.12285 (7)	0.0128 (2)	
O2	0.65505 (15)	0.73605 (8)	0.27723 (8)	0.0126 (2)	
O3	1.02939 (15)	0.66897 (8)	0.27336 (7)	0.01179 (19)	
O4	0.95582 (15)	0.87250 (8)	0.29863 (8)	0.01210 (19)	
O5	0.39485 (16)	0.59047 (8)	0.15004 (8)	0.0137 (2)	
O6	0.19308 (16)	0.40995 (8)	−0.00527 (8)	0.0135 (2)	
O7	0.63321 (15)	0.36233 (8)	0.10452 (8)	0.01286 (19)	
H5A	0.474 (3)	0.6368 (14)	0.1868 (14)	0.015*	
H6A	0.134 (3)	0.4002 (15)	0.0553 (13)	0.015*	
H7A	0.753 (3)	0.3688 (15)	0.1418 (14)	0.015*	
H5B	0.273 (2)	0.6050 (15)	0.1724 (14)	0.015*	
H6B	0.162 (3)	0.3514 (14)	−0.0441 (14)	0.015*	
H7B	0.548 (3)	0.3290 (15)	0.1482 (13)	0.015*	

### Atomic displacement parameters (Å^2^)

**Table:**

	*U*^11^	*U*^22^	*U*^33^	*U*^12^	*U*^13^	*U*^23^
Mn1	0.00931 (15)	0.00906 (15)	0.00839 (15)	−0.00039 (9)	0.00042 (10)	0.00021 (9)
S1	0.00802 (16)	0.00845 (16)	0.00839 (17)	0.00010 (10)	0.00033 (11)	−0.00019 (10)
N1	0.0120 (5)	0.0100 (5)	0.0103 (5)	0.0002 (4)	0.0001 (4)	−0.0003 (4)
N2	0.0109 (5)	0.0105 (5)	0.0102 (5)	−0.0002 (4)	0.0007 (4)	−0.0012 (4)
C1	0.0128 (6)	0.0120 (6)	0.0127 (6)	0.0003 (4)	−0.0010 (4)	0.0025 (5)
C2	0.0112 (6)	0.0119 (6)	0.0141 (6)	0.0008 (4)	−0.0006 (4)	0.0019 (5)
C3	0.0103 (6)	0.0089 (5)	0.0104 (6)	−0.0016 (5)	0.0015 (4)	0.0009 (4)
O1	0.0167 (5)	0.0125 (4)	0.0090 (4)	−0.0011 (3)	−0.0005 (3)	0.0010 (3)
O2	0.0088 (4)	0.0139 (4)	0.0150 (5)	−0.0014 (3)	0.0021 (3)	−0.0016 (3)
O3	0.0116 (4)	0.0115 (4)	0.0123 (4)	0.0028 (3)	0.0006 (3)	0.0016 (3)
O4	0.0124 (4)	0.0110 (4)	0.0129 (4)	−0.0018 (3)	0.0009 (3)	−0.0027 (3)
O5	0.0106 (4)	0.0166 (5)	0.0141 (5)	−0.0010 (4)	0.0026 (3)	−0.0052 (4)
O6	0.0139 (4)	0.0152 (5)	0.0116 (5)	−0.0036 (4)	0.0027 (3)	−0.0025 (4)
O7	0.0106 (4)	0.0145 (5)	0.0134 (5)	−0.0004 (3)	0.0002 (3)	0.0035 (3)

### Geometric parameters (Å, °)

**Table:**

Mn1—O6	2.1335 (10)	N2—C3	1.3371 (16)
Mn1—O6^i^	2.1335 (10)	N2—C2	1.3771 (17)
Mn1—O7^i^	2.1856 (10)	N2—H2A	0.8800
Mn1—O7	2.1856 (10)	C1—C2	1.3589 (19)
Mn1—O5	2.2218 (10)	C1—H1B	0.9500
Mn1—O5^i^	2.2218 (10)	C2—H2B	0.9500
S1—O1	1.4653 (10)	C3—C3^ii^	1.445 (2)
S1—O4	1.4759 (10)	O5—H5A	0.843 (14)
S1—O2	1.4839 (10)	O5—H5B	0.813 (14)
S1—O2	1.4839 (10)	O6—H6A	0.839 (14)
S1—O3	1.4989 (9)	O6—H6B	0.846 (14)
N1—C3	1.3296 (17)	O7—H7A	0.851 (14)
N1—C1	1.3754 (17)	O7—H7B	0.846 (14)
N1—H1A	0.8800		
			
O6—Mn1—O6^i^	180.0	C3—N1—C1	108.94 (11)
O6—Mn1—O7^i^	91.90 (4)	C3—N1—H1A	125.5
O6^i^—Mn1—O7^i^	88.10 (4)	C1—N1—H1A	125.5
O6—Mn1—O7	88.10 (4)	C3—N2—C2	108.33 (11)
O6^i^—Mn1—O7	91.90 (4)	C3—N2—H2A	125.8
O7^i^—Mn1—O7	180.0	C2—N2—H2A	125.8
O6—Mn1—O5	89.18 (4)	C2—C1—N1	106.82 (11)
O6^i^—Mn1—O5	90.82 (4)	C2—C1—H1B	126.6
O7^i^—Mn1—O5	91.53 (4)	N1—C1—H1B	126.6
O7—Mn1—O5	88.47 (4)	C1—C2—N2	107.28 (11)
O6—Mn1—O5^i^	90.82 (4)	C1—C2—H2B	126.4
O6^i^—Mn1—O5^i^	89.18 (4)	N2—C2—H2B	126.4
O7^i^—Mn1—O5^i^	88.47 (4)	N1—C3—N2	108.63 (11)
O7—Mn1—O5^i^	91.53 (4)	N1—C3—C3^ii^	125.62 (15)
O5—Mn1—O5^i^	180.0	N2—C3—C3^ii^	125.75 (15)
O1—S1—O4	110.58 (6)	Mn1—O5—H5A	124.6 (12)
O1—S1—O2	110.35 (6)	Mn1—O5—H5B	131.4 (13)
O4—S1—O2	109.94 (6)	H5A—O5—H5B	101.5 (17)
O1—S1—O2	110.35 (6)	Mn1—O6—H6A	115.7 (12)
O4—S1—O2	109.94 (6)	Mn1—O6—H6B	126.2 (12)
O1—S1—O3	109.47 (6)	H6A—O6—H6B	107.0 (17)
O4—S1—O3	109.23 (6)	Mn1—O7—H7A	123.0 (12)
O2—S1—O3	107.21 (6)	Mn1—O7—H7B	118.8 (12)
O2—S1—O3	107.21 (6)	H7A—O7—H7B	103.3 (17)
			
C3—N1—C1—C2	0.07 (15)	C1—N1—C3—C3^ii^	179.08 (15)
N1—C1—C2—N2	0.36 (14)	C2—N2—C3—N1	0.71 (14)
C3—N2—C2—C1	−0.66 (14)	C2—N2—C3—C3^ii^	−178.85 (15)
C1—N1—C3—N2	−0.48 (14)		

Symmetry codes: (i) −*x*+1, −*y*+1, −*z*;  (ii) −*x*, −*y*+2, −*z*.

### Hydrogen-bond geometry (Å, °)

**Table:**

*D*—H···*A*	*D*—H	H···*A*	*D*···*A*	*D*—H···*A*
N1—H1A···O3^iii^	0.88	1.93	2.7699 (15)	159
N1—H1A···O2^iii^	0.88	2.45	3.0994 (15)	131
N2—H2A···O3^iv^	0.88	1.92	2.7562 (16)	159
O5—H5A···O2	0.843 (14)	1.917 (15)	2.7600 (15)	176.8 (18)
O5—H5B···O3^v^	0.813 (14)	2.088 (15)	2.8638 (15)	159.7 (17)
O6—H6A···O4^vi^	0.839 (14)	1.908 (15)	2.7402 (15)	171.2 (18)
O6—H6B···O1^i^	0.846 (14)	1.847 (15)	2.6904 (14)	174.2 (18)
O7—H7A···O4^vii^	0.851 (14)	1.888 (15)	2.7298 (15)	169.5 (18)
O7—H7B···O2^vi^	0.846 (14)	1.892 (14)	2.7266 (14)	169.0 (17)

Symmetry codes: (iii) −*x*+1, *y*+1/2, −*z*+1/2; (iv) *x*−1, −*y*+3/2, *z*−1/2;  (v) *x*−1, *y*, *z*; (vi) −*x*+1, *y*−1/2, −*z*+1/2; (i) −*x*+1, −*y*+1, −*z*; (vii) −*x*+2, *y*−1/2, −*z*+1/2.
